# Roux-en-Y gastric bypass surgery changes fungal and bacterial microbiota in morbidly obese patients—A pilot study

**DOI:** 10.1371/journal.pone.0236936

**Published:** 2020-07-31

**Authors:** Robert E. Steinert, Ateequr Rehman, Everton Job Souto Lima, Valeria Agamennone, Frank H. J. Schuren, Daniel Gero, Phillip Schreiner, René Vonlanthen, Aiman Ismaeil, Stefanos Tzafos, Hanna Hosa, Diana Vetter, Benjamin Misselwitz, Marco Bueter

**Affiliations:** 1 Department of Surgery, University Hospital Zürich, Zürich, Switzerland; 2 Institute of Clinical Molecular Biology, Christian-Albrechts-University of Kiel, Kiel, Germany; 3 Microbiology and Systems Biology, The Netherlands Organization for Applied Scientific Research (TNO), Zeist, The Netherlands; 4 Department of Surgery, Aswan University, Tingar, Egypt; 5 Division of Visceral Surgery and Medicine, Inselspital Bern and Bern University, Bern, Switzerland; University of Illinois, UNITED STATES

## Abstract

The Roux-en-Y gastric bypass (RYGB) remains the most effective treatment for morbidly obese patients to lower body weight and improve glycemic control. There is recent evidence that the mycobiome (fungal microbiome) can aggravate disease severity in a number of diseases including inflammatory bowel disease (IBD), irritable bowel syndrome (IBS) and hepatitis; moreover, a dysbiotic fungal microbiota has been reported in the obese. We characterized fungal and bacterial microbial composition in fecal samples of 16 morbidly obese patients before and three months after RYGB surgery and compared with nine healthy controls. We found that RYGB surgery induced a clear alteration in structure and composition of the gut fungal and bacterial microbiota. Beta diversity analysis revealed significant differences in bacterial microbiota between obese patients before surgery and healthy controls (*P < 0*.*005*) and a significant, unidirectional shift in RYGB patients after surgery (*P < 0*.*001 vs*. *before surgery*). In contrast, there was no significant difference in fungal microbiota between groups but individually specific changes after RYGB surgery. Interestingly, RYGB surgery induced a significant reduction in fungal alpha diversity namely Chao1, Sobs, and Shannon diversity index (*P<0*.*05*, *respectively*) which contrasts the trend for uniform changes in bacteria towards increased richness and diversity post-surgery. We did not observe any inter-kingdom relations in RYGB patients but in the healthy control cohort and there were several correlations between fungi and bacteria and clinical parameters (*P<0*.*05*, *respectively*) that warrant further research. Our study identifies changes in intestinal fungal communities in RYGB patients that are distinct to changes in the bacterial microbiota.

## Introduction

The Roux-en-Y gastric bypass (RYGB) remains the most effective treatment for morbidly obese patients to lower body weight and improve glycemic control [1]. Several interrelated mechanism have been suggested for this including alterations in the postprandial secretion of gastrointestinal hormones [2, 3], increased delivery of bile acid salts to distal L-cells [2, 4], faster gastric transit and changes in vagal nerve signaling [5] and, more recently, changes in gut microbiota composition [6]. The gut microbiota is a dense and highly diverse microbial community composed of bacteria, fungi, phages, viruses, protozoa, and archaea that inhabits the gastrointestinal tract and is intimately involved in host physiology [7, 8]. An impairment of the gut microbiota, referred to as ‘dysbiosis’ and characterized by changes in its composition and function, is commonly observed in gastrointestinal and systemic diseases [9, 10]. In the obese, bacterial dysbiosis is characterized by compositional changes in specific bacterial groups and low bacterial gene richness and related functional pathways [11–13]. RYGB surgery restores bacterial richness and diversity and substantially alters the abundance of several bacterial groups [14–16]. It has been speculated that these alterations may support weight loss and metabolic improvements seen after surgery.

The fungal components of the gut microbiota have hardly been explored in health and disease. Fungi comprise less than 2% of total gut microorganisms, with only about 60 genera and 180 species [17]. A fungal cell is, however, about >100-fold larger than a bacterial cell and thus, represents substantially greater biomass than suggested by the number of available genomes [17]. Mycobiome dysbiosis has recently been implicated in the pathogenesis of inflammatory bowel diseases (IBD) [18], irritable bowel syndrome (IBS) [19] and hepatitis [20] and Rodríguez *et al*. [21] observed an increased abundance of *Ascomycota*, *Saccharomycetes*, *Dipodascaceae* and *Saccharomycetaceae* in the obese [21]. To date, it has not been explored whether RYGB surgery has an impact on fungal microbiota and whether potential changes are related to the metabolic improvements postoperatively.

Here we used an amplicon-based barcoding approach to characterize fungal and bacterial microbiota in fecal samples of morbidly obese patients before and three months after RYGB surgery and healthy, normal-weight controls. We applied a variety of analyses to assess differences in microbial community composition, possible inter-kingdom correlations, and relationships with clinical patient parameters.

## Materials and methods

### Patients and sample collection

Sixteen RYGB patients were recruited at the Department of Surgery, University Hospital Zurich, Switzerland. Each participant gave written, informed consent before participating in the study. All patients underwent complete evaluation before the operation and during follow-up, including medications, nutritional behavior, anthropometric and clinical parameters, and blood sampling for laboratory tests. RYGB patients received surgery between July and September 2017. Fecal samples were collected the day before surgery and three months after, using the deoxyribose nucleic acid (DNA) Genotek Omnigene Gut Collection kits (DNA Genotek, Ontario, Canada) following the manufacturer’s instructions. The kits were returned to a −80 °C freezer within 24 h of collection and stored until analysis.

A separate cohort of Dutch healthy normal weight subjects was used as a baseline. This approach was implied to see the direction of microbial changes after RYGB surgery towards, or against a healthy microbiome profile. Normal-weight controls were nine non-smokers, non-drug using individuals from the Netherlands who did not regularly experience intestinal problems and were not under treatment for conditions or diseases of the digestive tract. Healthy subjects had not taken antibiotics for at least seven months before sampling, nor had they used other medications in the previous two weeks. Each participant gave written, informed consent before participating in the study.

Subject characteristics are presented in Table 1. All research was carried out in compliance with the Helsinki Declaration, and for the Swiss RYGB cohort, it was approved by the ethics committee Zurich, Switzerland (BASEC Nr. 2012–0260) while for the healthy Dutch controls it was approved by the ethics committee Brabant, The Netherlands.

**Table 1:** Subject characteristics.

	Healthy controls (n = 9)	RYGB patients (before surgery, n = 16)	RYGB patients (3 month after surgery, n = 16)
**Age (yr)**	49.8 ± 5.0 (22–64)^a^	39.8 ± 3.1 (22–67)^a^	39.8 ± 3.1 (22–67)^a^
**Gender (m/f)**	5/4	5/11	5/11
**Weight (kg)**	75.0 ± 4.3 (60–95)^a^	119 ± 6.0 (93–169)^b^	96.9 ± 5.4 (78–136)^c^
**BMI (kg/m2)**	22.5 ± 0.8 (20.3–26.9)^a^	41.6 ± 1.4 (35.6–51.9)^b^	33.9 ± 1.4 (24.3–43.7)^c^
**Hip-to-Waist Ratio**	-	1.1 ± 0.1 (0.9–1.2)^a^	1.1 ± 0.1 (0.9–1.3)^a^
**Hemoglobin A1c (%)**	-	5.5 ± 0.1 (5.1–6.2)^a^	5.1 ± 0.1 (4.8–5.5)^b^
**Fasting glucose (mmol/L)**	-	5.7 ± 0.2 (4.4–7.2)^a^	4.6 ± 0.1 (3.6–5.4)^b^
**Total cholesterol (mmol/l)**	-	4.4 ± 0.2 (2.9–6.1)^a^	3.7 ± 0.2 (2.7–5.0)^b^
**HDL-cholesterol (mmol/l**	-	1.1 ± 0.2 (0.8–1.5)^a^	1.2 ± 0.1 (0.7–1.6)^a^
**LDL-cholesterol (mmol/l)**	-	2.7 ± 0.2 (1.8–4.1)^a^	2.0 ± 0.1 (1.1–2.8)^b^
**Triglyceride (mmol/l)**	-	1.7 ± 0.3 (0.7–5.4)^a^	1.1 ± 0.1 (0.7–1.8)^a^
**C-reactive protein (mg/L)**	-	6.6 ± 1.2 (0.7–19.0)^a^	4.5 ± 1.3 (0.8–20.0)^a^

BMI, body mass index; HDL, high-density-lipoprotein; LDL, low-density lipoprotein; Data presented as mean ± standard error of mean, with ranges in parentheses. Means with different letter superscripts differ significantly between groups, P<0.05. To test for significant differences between groups, comparisons used Student’s independent and paired t-test.

### DNA isolation, amplicon PCR and illumina sequencing

DNA was extracted from fecal samples, as described previously [22]. DNA samples were then used for the 16S rRNA gene and Internal Transcribed Spacer (**ITS**) region sequencing to characterize bacterial and fungal communities (for details, see S1 File).

### Microbiome (16S and ITS) data analysis

The 16S rRNA gene and ITS sequencing data were pre-processed, analyzed, and classified using Mothur (v. 1.36.1) [23]. Low-quality regions of the 16S rRNA gene sequences were trimmed using Btrim [24] with a sliding window size of 5 nucleotide and an average quality score of 25. For the ITS sequences, cutadapt was used for trimming [25] with a quality cutoff of 20. Paired-end reads were merged, and unique sequences were filtered based on length, with no ambiguous bases allowed. Chimeric sequences were identified and removed using UCHIME (Edgar 2011). Unique 16S rRNA gene sequences were aligned to the bacterial SILVA SEED reference database (Release 128, available at https://mothur.org/wiki/Silva_reference_files). Taxonomic assignment was performed using the Ribosomal Database Project (RDP) naïve Bayesian classifier with a confidence threshold of 60% and 1000 iterations [26] and the mothur-formatted version of the RDP training set v.14 (trainset14_032015). Unique ITS sequences were classified using the UNITE reference database v.6 (mothur release, available at https://unite.ut.ee/repository.php), with a confidence threshold of 60% and 1000 iterations. 16S rRNA gene and ITS sequence data have been submitted to the European Nucleotide Archive (accession no. PRJEB38917).

### Statistical analysis

Statistical analysis was conducted using the open source software R, version 3.5.1 [27], and figures were generated using ggplot2 [28]. Descriptive statistics were used for demographic variables of the RYGB and heathy control cohort. To test for significant differences between groups, comparisons used Student’s independent and paired t-test. Differences in bacterial and fungal community composition between the sample groups were assessed at the phylum, class, family and genus level using the DESeq2 package (Love 2014). The Benjamini-Hochberg (BH) false discovery rate (FDR) correction was applied to the resulting p-values [29]. Microbial alpha diversity estimations are known to be influenced by sequencing depth. Thus, to rarify this uneven sequencing depths, subsampling (bacterial 11514 seqeunces per samples and mycobiome 98 sequences per sample) approach was implemented to calculate four diversity indices.: (i) Abundance-based Coverage Estimator (ACE) metric, (ii) Chao1, (iii) observed species, and (iv) Shannon’s diversity. Analysis of variance (ANOVA) was performed to test global differences in diversity among groups, in addition, paired t-tests was applied to compare RYGB patinets before and after surgery. For beta diversity analysis, due to uneven sampling depth (library size), the microbiome data was normalized using the variance stabilizing transformation (VST) method in the DESeq2 package. The transformed data was analyzed with PerMANOVA using the adonis function of the package vegan in R (http://cc.oulu.fi/~jarioksa/softhelp/vegan/html/adonis.html) to determine community composition variation across groups at different taxonomic levels. P-value adjustment was done via p.adjust function using the Benjamini & Hochberg method. Spearman correlation analysis was used to explore the relationship between changes in bacterial and fungal communities, and between these changes and clinical patient parameters. The Benjamin-Hochberg method was used for adjustment of p-values [29]. Ordination analysis was performed using Non-metric Multidimensional Scaling (NMDS) to investigate patterns in community composition.

## Results

### RYGB surgery changes mycobiome diversity

**Fig 1:**
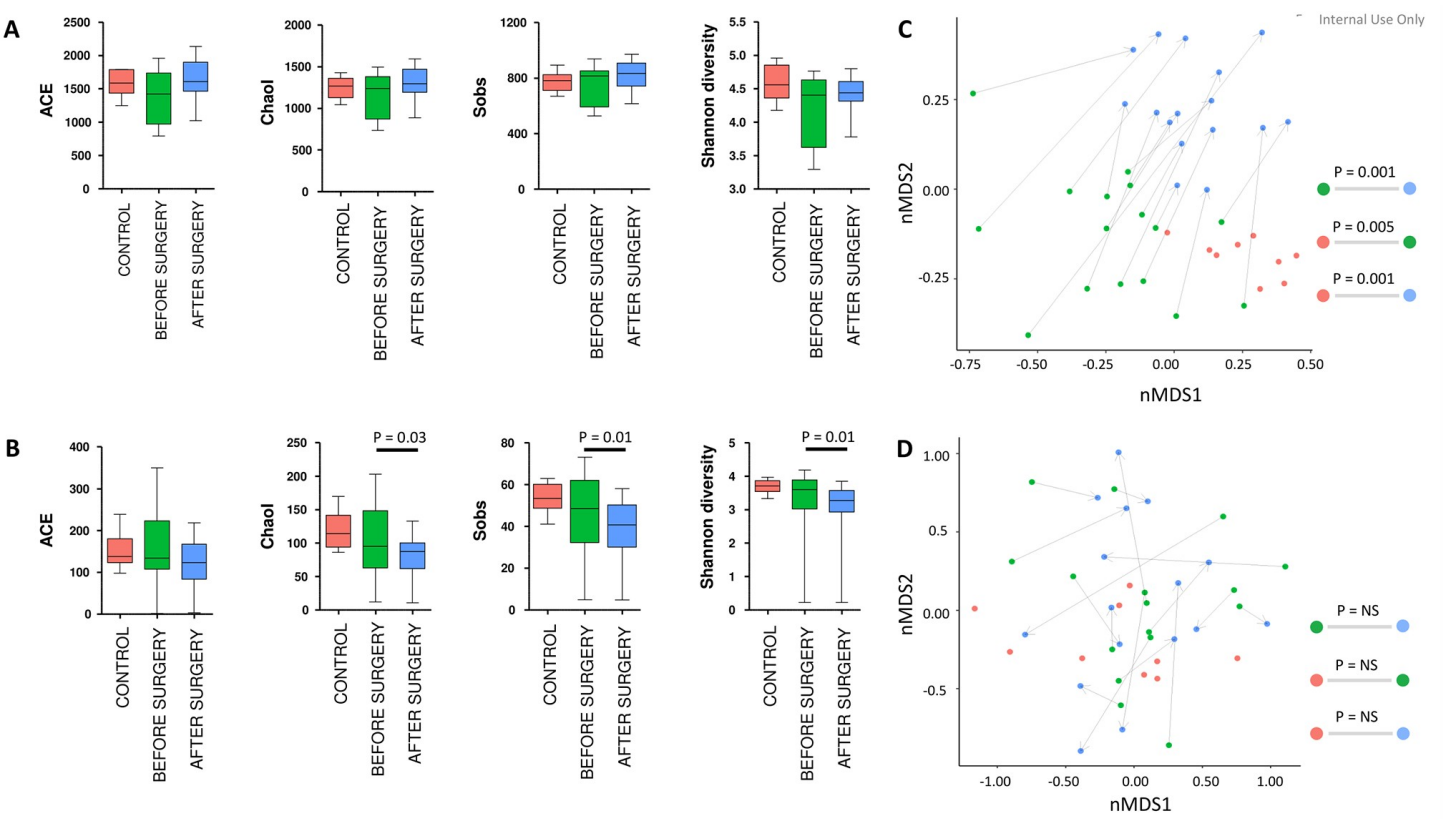
Alpha diversity of observed species of bacterial (**A**) and fungal (**B**) microbiota composition in patients before and after RYGB surgery and the healthy control cohort. Analysis of variance (ANOVA) was performed to test global differences in diversity among groups, in addition, paired t-tests was applied to compare RYGB patients before and after surgery. Non-metric Multi Dimensional Scaling (NMDS) plots were generated using Bray-Curtis distances (Beta diversity) for bacterial (**C**) and fungal (**D**) microbiota genera. Data were analyzed with PerMANOVA to determine community composition variation across groups at different taxonomic levels. P-value adjustment was done via p.adjust function using the Benjamini & Hochberg method.

Bacterial and fungal alpha-diversity was comparable between in RYGB patients before surgery and the healthy control cohort (Fig 1A and 1B). After surgery, a trend for an increase in bacterial alpha-diversity was observed when compared to patients before surgery in measures of estimated alpha-diversity (ACE and Chao1). There was, however, no noticeable change in the number of observed bacterial species (Sobs) and Shannon diversity (Fig 1A). In contrast, for fungi we observed a significant reduction in estimated (Chao1, *P = 0*.*03*) and observed (Sobs, *P = 0*.*01*) species richness and Shannon diversity (*P = 0*.*01*, Fig 1B).

For bacterial composition, PerMANOVA suggested significant differences between RYGB patients before surgery and the healthy control cohort (*P = 0*.*005*). The effect of surgery on microbial composition and structure was very distinct and unidirectionally resulting in significant differences between patients before and after surgery (*P = 0*.*001*). Patients after surgery were, however, still distinct from the healthy control cohort (*P = 0*.*001*, Fig 1C). For fungal microbiota, NMDS analysis revealed no differences between RYGB patients before surgery and the control cohort. However, in patients after surgery, we observed a clear alteration in composition and structure when compared to before surgery that in contrast to the homogeneous shift in bacterial beta-diversity was specific to individuals and, therefore, not significantly different between groups (Fig 1D).

### Bacterial microbiota composition changes after surgery

**Fig 2:**
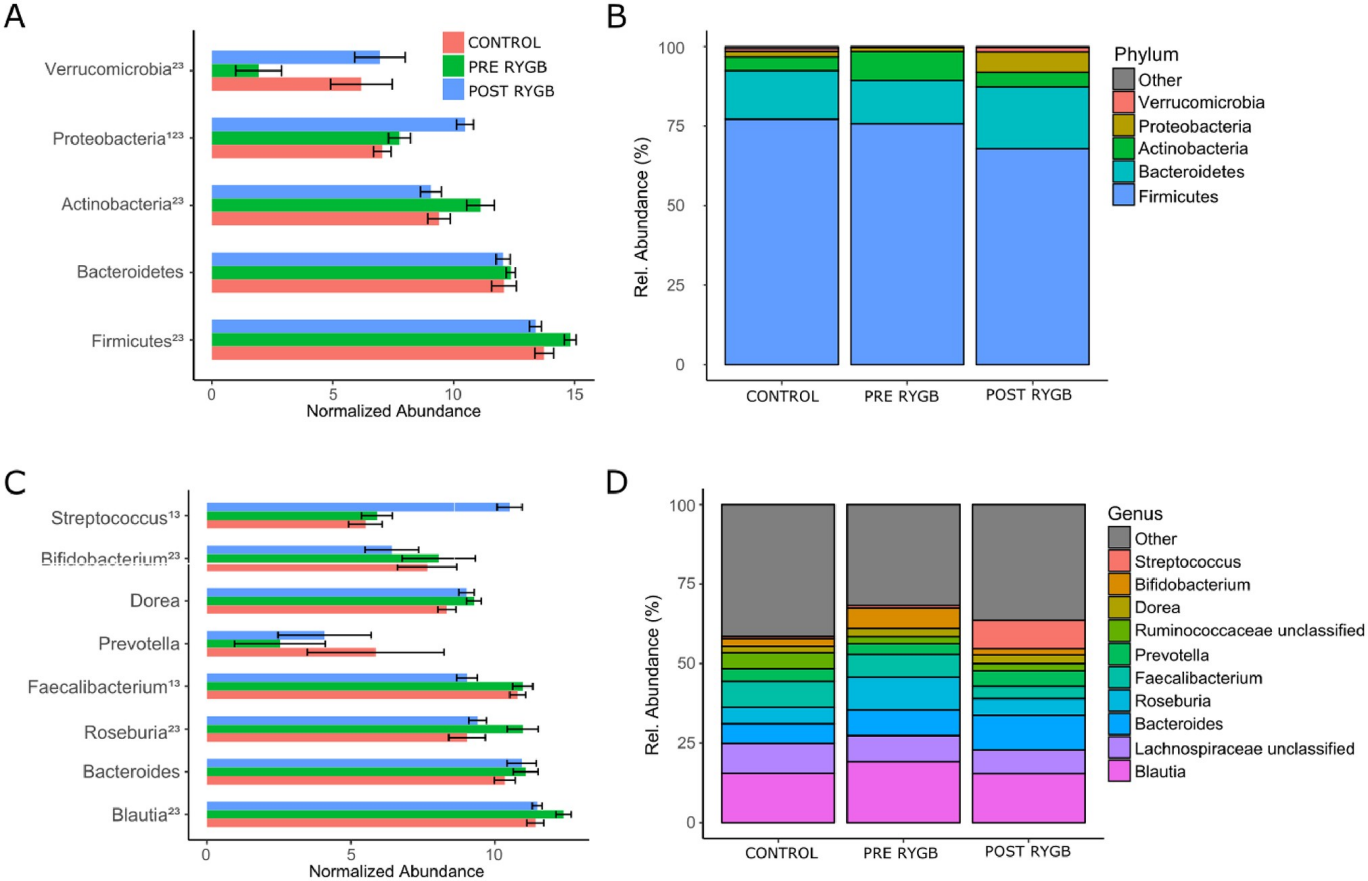
Normalized mean abundance ± SEM (left) and relative abundance counts (right) for major bacteria at phylum (A and B) and genus (C and D) level in RYGB patients before and 3 month after surgery and in healthy controls. Minor bacteria are grouped under ‘Other’. Differential abundance test was done by DESeq2 and considered to be significant at p <0.05, where ^(1)^ indicates differentially between control and after surgery, ^(2)^ between control and before surgery, and ^(3)^ between before surgery and after surgery.

The microbiota of RYGB patients and normal-weight controls was dominated by *Firmicutes*, specifically by members of the *Clostridia* class. Several significant differences in normalized abundance at all taxonomical levels were observed between groups (Fig 2 and S1 Fig). At the phylum level, pre-surgery patients had significantly higher levels of *Firmicutes* and *Actinobacteria* and lower levels of *Verrucomicrobia* when compared to healthy controls (*P ≤ 0*.*05*, *respectively*). Post-surgery, abundances of these bacterial groups were not significantly different anymore, indicating a rebiosis of microbiota (Fig 2A and 2B). *Proteobacteria* levels were significantly different between all groups (*P ≤ 0*.*05*, *respectively*) with healthy controls showing the lowest levels and patients after surgery the highest (Fig 2A and 2B). At the genus level, patients before surgery had significantly higher levels of *Blautia* and *Roseburia* when compared with healthy controls (*P ≤ 0*.*05*, *respectively*) which decreased post-surgery in the direction of a healthy microbiome (Fig 2C and 2D). Abundances of *Faecalibacterium* and *Bifidobacterium* also decreased after surgery. However, this decrease was beyond abundances observed in healthy controls (Fig 2C and 2D). In line with observations at family and class level (S1 Fig), aerotolerant *Streptococcus* was significantly increased after surgery (*P ≤ 0*.*05*, *respectively*).

### RYGB induces fungal microbiota changes at individual level

**Fig 3:**
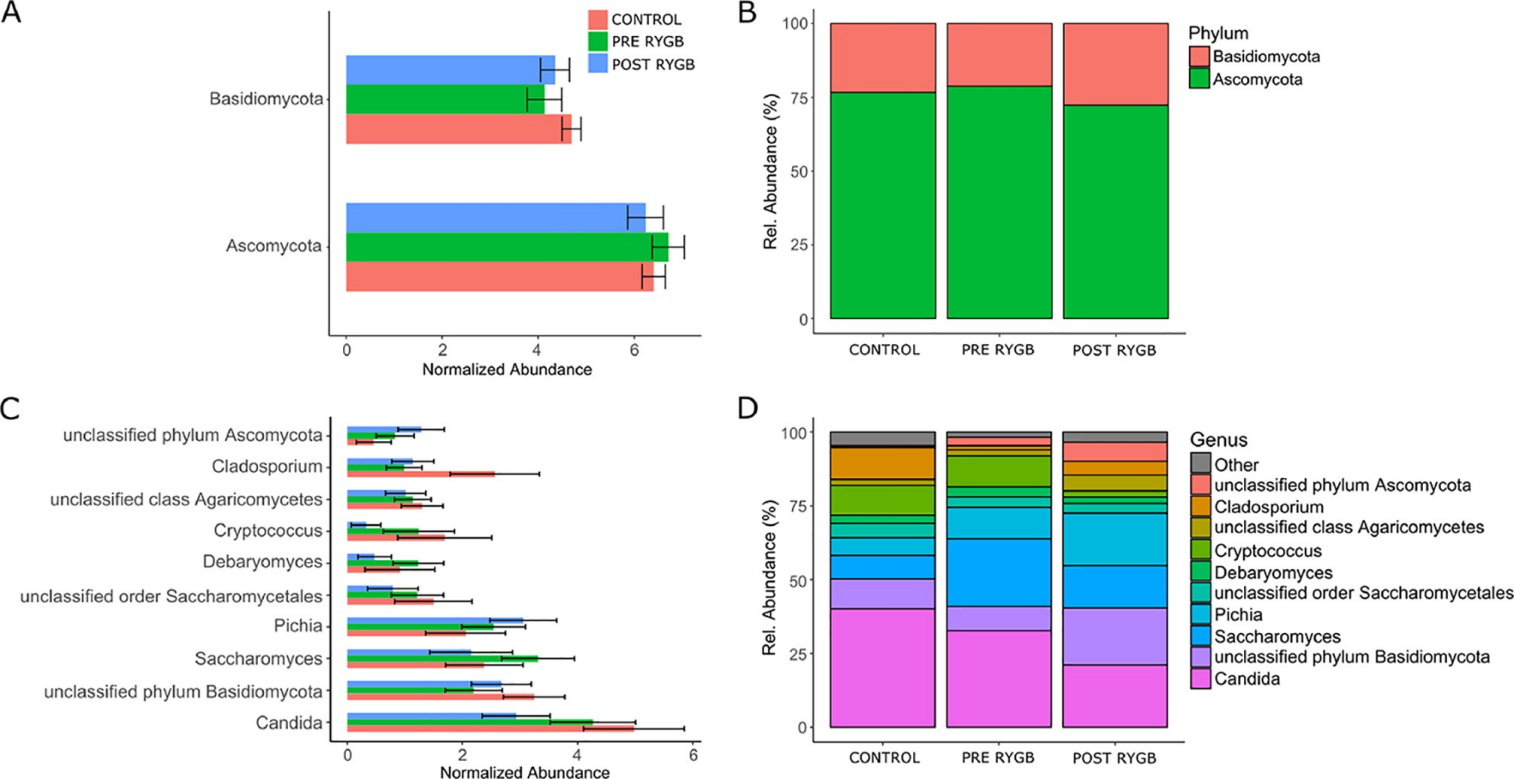
Normalized mean abundance ± SEM (left) and relative abundance counts (right) for major fungi at phylum (A and B) and genus (C and D) level in RYGB patients before and 3 month after surgery and in healthy controls. Minor fungi are grouped under ‘Other’. There were no significant differences between subject groups.

As mentioned, changes in fungal microbiota composition in response RYGB surgery were observed on an individual level. There was, however, no unidirectional change towards, or against, a healthy microbiome (Fig 1). This effect resulted in a lack of significant changes in normalized abundance at all taxonomical levels (Fig 3 and S2 Fig). *Ascomycota* and *Basidiomycota* were the most dominant fungal phyla in all subjects, and RYGB surgery induced a change of these fungal groups towards a healthy microbial profile (Fig 3A and 3B). C*andida*, *Saccharomyces*, and *Pichia* were among the most abundant fungi at the genus level with decreases in *Candida* and *Saccharomyces* and increases in *Pichia* after surgery (Fig 3C and 3D). When compared to healthy controls, changes in *Saccharomyces* were towards, and changes in *Candida* and *Pichia*, against, a healthy mycobiome. *Cladosporium* was less abundant in patients before surgery when compared with healthy controls. However, surgery increased its abundances towards a healthy mycobiome. In contrast, *Cryptococcus* was observed to be less abundant before surgery when compared with healthy controls, and this trend increased after surgery (Fig 3C and 3D).

### Associations between fungal and bacterial microbiota and clinical parameters

**Fig 4:**
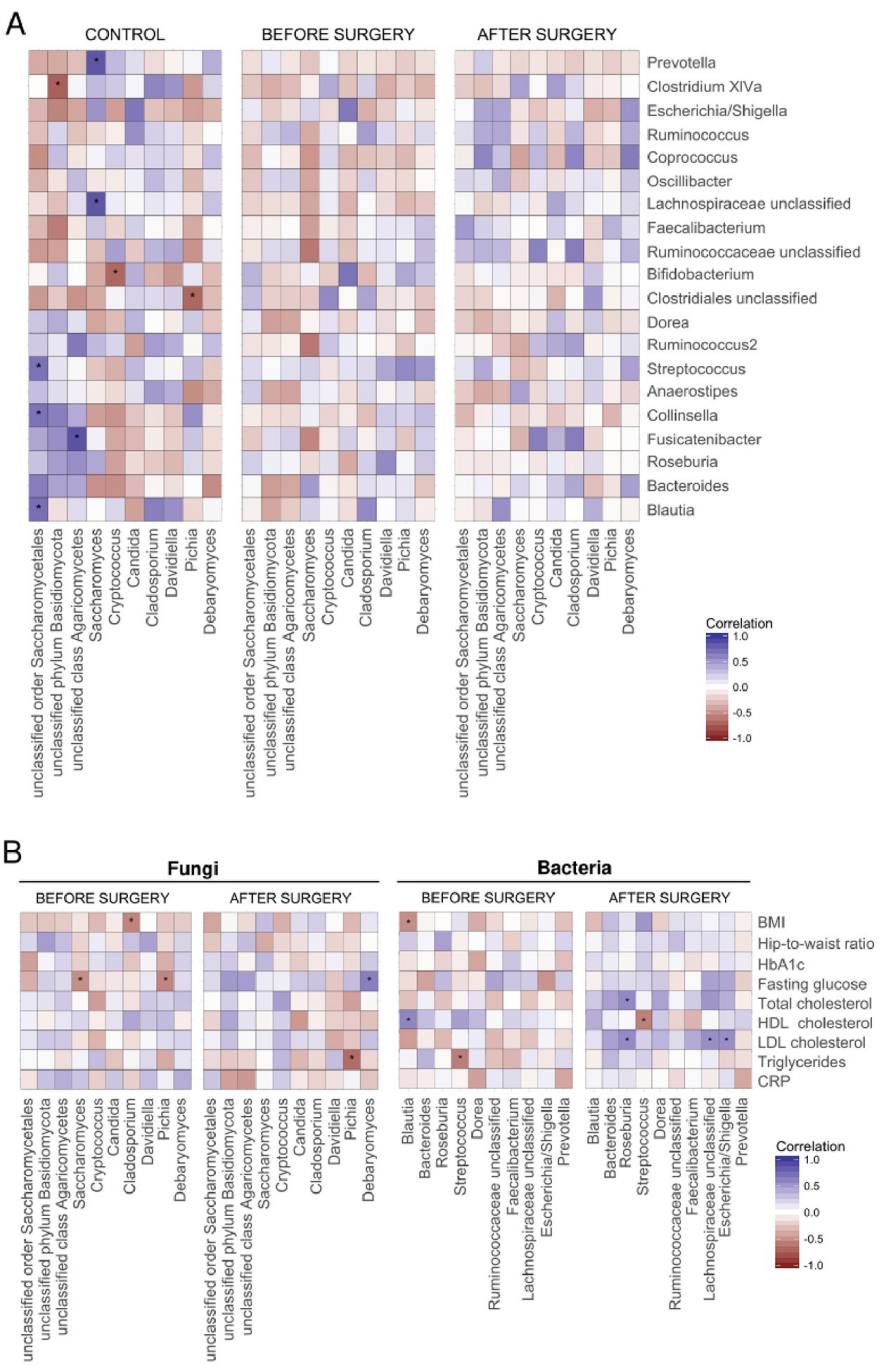
Heatmap of Spearman’s pairwise correlation coefficients between fungi and bacterial microbiota at genus level (A) and with metabolic parameters before and after surgery (B). Only the top 20 abundant bacterial and top 8 fungi are shown. Blue squares indicate positive correlations, and red squares indicate inverse correlations. The shading of squares indicates the magnitude of the association; darker shades are more strongly associated than lighter shades. P < 0.05 significance denoted by *. BMI, body mass index; HbA1c, haemoglobin A1c; HDL, high-density-lipoprotein; LDL, low-density lipoprotein, CRP, C-reactive protein.

To further understand the interactions between bacterial and fungal groups in healthy subjects and RYGB patients, correlation analysis was performed in all three study groups separately. In healthy subjects, we found six positive correlations (Fig 4A) and three negative correlations while we failed to identify such inter-kingdom correlation in RYGB subjects before and after surgery.

We also identified several significant correlations between fungal and bacterial genera and clinical patient parameters before and after surgery (Fig 4B). For fungal genera before surgery, *Saccharomyces* and *Pichia* correlated negatively with fasting glucose and *Cladosporium* negatively with BMI. After surgery, we lost these correlations but identified a negative correlation between *Pichia* and triglycerides and a positive correlation between *Debaryomyces* and fasting glucose (Fig 4B). For bacterial genera before surgery, *Blautia* correlated positively with HDL cholesterol and negatively with BMI, while *Streptococcus* correlated negatively with triglycerides. After surgery, we were again unable to find any of those correlations. However, there were positive correlations between *Roseburia* and cholesterol (total and LDL), as well as between one *unclassified Lachnospiraceae* bacterium and *Escherichia/Shigella* and LDL cholesterol. Furthermore, *Streptococcus* correlated negatively with HDL cholesterol (Fig 4B).

## Discussion

We characterized fungal and bacterial microbial composition in fecal samples of morbidly obese patients before and 3 months after RYGB surgery and compared with a healthy control cohort. We found clear but highly individualized changes in the fungal kingdom with a significant reduction in mycobiome richness and diversity in patients after surgery when compared to before surgery. This effect strikingly contrasts the well-described and uniform changes in bacteria towards increased richness and diversity post-surgery.

Consistent with many other studies, we found the gut mycobiome was dominated by fungi from the *Ascomycota* and *Basidiomycota* phyla [18, 19, 21] while *Candida*, *Saccharomyces*, and *Cladosporium* were the main genera [30–32]. *Ascomycota* appeared more prevalent in obese patients before surgery than after surgery and normal-weight controls while at genus level similar trends were observed for *Saccharomyces* and *Debaryomyces*. These observations are in line with a previous study comparing the fungal microbiome of 52 obese and non-obese patients [21], possibly indicating an effect of the obese phenotype on fungal microbiota composition. We also found a tendency for higher levels of *Candida* in obese patients before surgery than after surgery supporting a link of *Candida* with metabolic changes and intestinal inflammation as previously observed for diabetes [33] and IBD [34].

There was no significant difference in fungal alpha-diversity between obese patients before surgery and the healthy control cohort used in this study. Other studies in patients with IBD and chronic hepatitis showed increased alpha-diversity when compared with healthy subjects [20, 35, 36] while one recent study also observed reduced alpha-diversity in IBD patients [18]. Rodríguez *et al*. [21] observed no significant difference in fungal alpha-diversity between obese and non-obese subjects which is consistent with our findings. Interestingly, we found that RYGB surgery resulted in a significant reduction in observed and estimated fungal richness and diversity. This clearly contrasts the many reports of increased bacterial alpha-diversity after RYGB surgery [14–16, 37–39] although in the current study we were not able to replicate these earlier observations but only observed a trend for increased alpha-diversity measures (ACE and Chao1). Perhaps this is due to the limited number of subjects in our study (n = 16) when compared to earlier studies (n = 31 and 61) [14, 37], and/or different sample times after surgery. In fact, it is likely that the gut microbiota still changes between 3 month and 1 year after surgery to dynamically adapt to alterations in host metabolism (weight loss, fat mass loss, etc.). Thus, further investigation is warranted to better understand changes in bacterial and fungal microbiome over time and whether there are opposite changes of fungal and bacterial diversity in relation to host disease and pathogenesis.

With regard to the bacterial microbiota, we found the typical increase in the relative abundance of *Proteobacteria* after surgery [14–16, 37–39]. While due to their proinflammatory properties *Proteobacteria* are generally not considered to be beneficial [40, 41], there was no increase in CRP levels after surgery in our cohort (Table 1). This *Proteobacteria* increase might reflect the increase in oxygen availability in the large intestine after surgery, favoring facultative anaerobes such as *Escherichia*, *Klebsiella*, and *Pseudomonas* [42, 43]. Along the same lines, we observed a decrease in obligatory anaerobic gram-positive bacterial groups such as *Blautia*, *Roseburia and Faecalibacterium* from the phylum *Firmicutes* as well as *Bifidobacterium* from the phylum *Actinobacteria* [15, 16, 39]. It is possible that these compositional changes are also linked to (i) reduced gastric acid secretion after surgery [44], (ii) reduction in total energy intake with modifications in nutrient composition and (iii) increased availability of undigested nutrients in the large intestine due to the anatomic alteration after surgery.

Interestingly, RYGB surgery resulted in individualized changes in the fungal microbiota and not in unidirectional changes, as observed for bacterial microbiota. One explanation is that fungi are aerobic and thus less sensitive to the increase in oxygen availability after surgery. This may limit unidirectional changes as seen with bacteria that are highly sensitive to such gastrointestinal conditions. Moreover, while it is known that eating habits can influence the bacterial gut microbiota, there may be an even stronger influence of daily diet on the gut mycobiome. This is supported by a study by Hallen-Adams *et al*. [45], which analyzed the composition of the gut mycobiome at two time points each from 24 participants and found detection of the same fungus occurred in less than 20% of the time. Also David *et al*. [46] conducted a controlled diet study and found while bacterial composition showed a clear response to nutrient availability (carbohydrates/fiber vs. proteins and fats), fungal composition appeared to be driven by food colonization with the same species of fungi detected in participant fecal samples and in cheese fed to those participants. In fact, sequencing methods do not distinguish between DNA contributed from live or dead cells. Thus, it is not possible to differentiate fungi colonizing the gut from transients derived from diet and/or environment. Among the most dominant genera in our cohorts were *Saccharomyces and Debaryomyces* which are both commonly found in foods such as mushrooms, cheese, bread and beer.

We also performed correlation analyses to identify inter-kingdom relations and associations of bacterial and fungal microbiota with anthropometric and metabolic parameters. Several studies suggested a direct interaction of fungi and bacteria. In IBD, Sokol *et al*. [18] reported disease-specific patterns for inter-kingdom networks with increased strenghts of correlations between fungi and bacteria in ulcerative colitis but weaker correlations in Crohn´s disease. We observed several significant correlations between bacteria and fungi in healthy, normal-weight controls but not in RYGB patients, which may indicate persistence of a dysbiotic microbiota state in the obese before and after surgery. Moreover, we found that *Pichia* was negatively correlated with fasting glucose before surgery and with triglycerides after surgery, while *Streptococcus* was negatively correlated with triglycerides before surgery and with HDL after surgery. These metabolic and microbial changes might be independent results of the surgical intervention. However, it warrants further research to investigate if the correlated fungal and bacterial groups might also directly modulate metabolic parameters independent of surgery. Such direct effects could be tested in a RYGB rat model with a targeted fungicide/antibiotic intervention.

There are some limitations of our study. 1) In this pilot study, we only included 16 RYGB patients and 9 healthy control subject, thus, whether our data are representative of larger cohorts remains to be proven. 2) The healthy subject cohort which was used as a baseline to interpret the direction of changes in our main patient cohort was sampled at a distinct place using a different sampling method and was not adjusted for age and gender. Since geography including different eating habits, sampling methodology, age and gender can all impact microbiome measurements, caution is required when directly comparing these two cohorts. 3) Fungal and bacterial microbial composition was characterized only before and three months after surgery. While this allows to better understand the immediate effects of surgery, it does not provide information on the dynamic changes that may occur later in the postoperative phase as an adaption to changes in host metabolism such as weight loss, fat mass loss, etc. 4)) All current sequencing technologies only asses DNA signatures and thus cannot distinguish between DNA contributed from colonizing cells or fungi or transients derived from the diet. 5) Associations between specific fungi and 3-month clinical data such as cholesterol values need to be confirmed with a longer follow-up.

In conclusion, we found a clear alteration in structure and composition of fungal and bacterial microbiota in response to RYGB surgery. Beta diversity analysis revealed significant differences in bacterial microbiota between obese patients before surgery and healthy controls (*P < 0*.*005*) and a significant, unidirectional shift in RYGB patients after surgery (*P < 0*.*001 vs*. *before surgery*). In contrast, there was no significant difference in fungal microbiota between groups but individually specific changes after RYGB surgery. Interestingly, RYGB surgery induced a significant reduction in fungal alpha diversity namely Chao1, Sobs, and Shannon diversity index (*P<0*.*05*, *respectively*) which contrasts the trend for uniform changes in bacteria towards increased richness and diversity post-surgery. We did not observe any inter-kingdom relations in RYGB patients but in the healthy control cohort and there were several correlations between fungi and bacteria and clinical parameters (*P<0*.*05*, *respectively*) that warrant further research.

## Supporting information

S1 File Supplementary material and methods. (DOCX)

S1 Fig Normalized mean abundance +/- SEM (left) and relative abundance counts (right) for major bacteria at class (A and B) and family (C and D) level in RYGB patients before and 3 month after surgery and in healthy controls. Minor bacteria are grouped under ‘Other’. Differential abundance test was done by DESeq2 and considered to be significant at p <0.05, where (1) indicates differentially between control and after surgery, (2) between control and before surgery, and (3) between before surgery and after surgery.
(TIFF)

S2 Fig Normalized mean abundance +/- SEM (left) and relative abundance counts (right) for major fungi at class (A and B) and family (C and D) level in RYGB patients before and 3 month after surgery and in healthy controls. Minor fungi are grouped under ‘Other’. There were no significant differences between subject groups.
(TIFF)
