# Supplementary material for: Roux-en-Y gastric bypass surgery changes fungal and bacterial microbiota in morbidly obese patients—A pilot study
Source: PLoS One. 2020 Jul 31;15(7):e0236936. doi: 10.1371/journal.pone.0236936 (PMC7394366; doi:10.1371/journal.pone.0236936)
Supplement: S1 File — (DOCX) [file pone.0236936.s001.docx]

***DNA isolation, amplicon PCR and Illumina sequencing***

The primers F515 and R806 (F515: 5′- TAT GGT AAT TGT GTG CCA GCM GCC GCG GTA A -3′; R806: 5′- AGT CAG TCA GCC GGA CTA CHV GGG TWT CTA AT -3’) were used to target the V4 region of the bacterial 16S rRNA gene (47), and the primers BITS and B58S3 (BITS: 5′- ACC TGC GGA RGG ATC A -3′; B58S3: 5′- GAG ATC CRT TGY TRA AAG TT -3′) (48) were used to target the ITS1 region of the fungal DNA. The composite 16S rRNA gene specific primers included Illumina adapters and a unique 8-nucleotide sample index sequence key (47). The PCR to obtain 16S rRNA gene and ITS amplicons was performed using the Phusion High-Fidelity PCR Master mix (New England Biolabs, Ipswich, MA, USA) with 100 pg template DNA. For the 16S rRNA gene, the following amplification program was used: initial denaturation for 30 s at 98 °C; 30 amplification cycles (10 s at 98 °C, 30 s at 55 °C and 30 s at 72 °C); and final elongation for 5 min at 72 °C. For the ITS, the following amplification program was used: initial denaturation for 30 s at 98 °C; 30 amplification cycles (10 s at 98 °C, 30 s at 48 °C and 30 s at 72 °C); and final elongation for 5 min at 72 °C. Adapters and index sequence keys were added to the ITS amplicons using the Nextera DNA Sample Preparation Kit (Illumina Technologies, San Diego, CA, USA). PCR products were analyzed on a Fragment Analyzer (Advanced Analytical Technologies Inc., Ankeny, IA, USA) to estimate DNA concentration. The amplicon libraries were then pooled in equimolar amounts and purified using first the QIAquick Gel Extraction Kit (Qiagen, Valencia, CA, USA) and then Agencourt AMPure magnetic beads (Beckman Coulter, Brea, CA, USA). DNA concentration of the pool was measured on a fluorometer using the Quant-iT PicoGreen dsDNA Assay Kit (Thermo Fisher Scientific, Waltham, MA, USA). Amplicon sequencing was performed on the Illumina MiSeq platform (Illumina Technologies, San Diego, CA, USA) using 2 x 250 cycle paired-end settings.
