# Supplementary figures and images for: Roux-en-Y gastric bypass surgery changes fungal and bacterial microbiota in morbidly obese patients—A pilot study
Source: PLoS One. 2020 Jul 31;15(7):e0236936. doi: 10.1371/journal.pone.0236936 (PMC7394366; doi:10.1371/journal.pone.0236936)

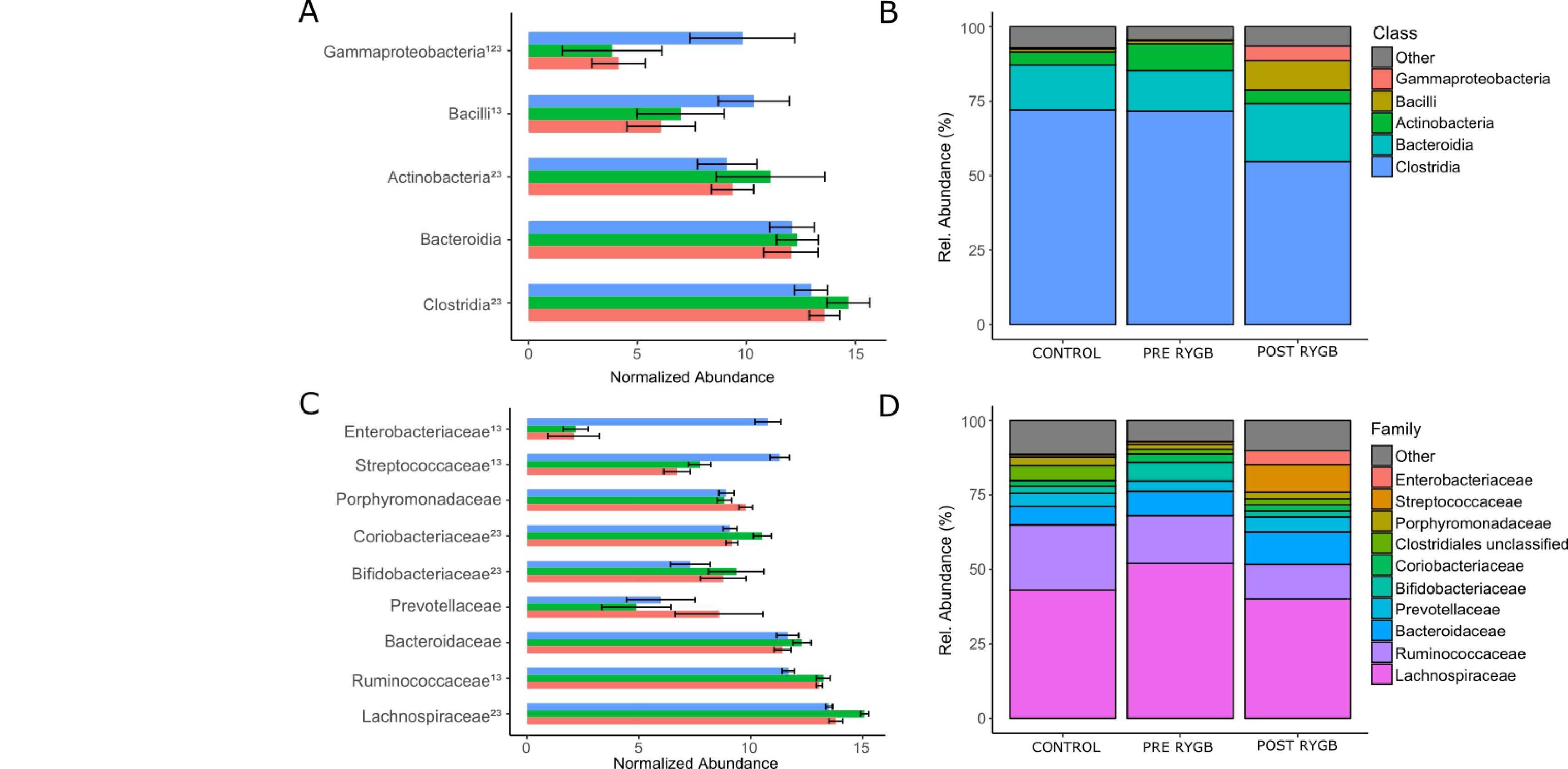

Supplement: S1 Fig — Minor bacteria are grouped under ‘Other’. Differential abundance test was done by DESeq2 and considered to be significant at p <0.05, where (1) indicates differentially between control and after surgery, (2) between control and before surgery, and (3) between before surgery and after surgery. (TIFF) [file pone.0236936.s002.tiff]

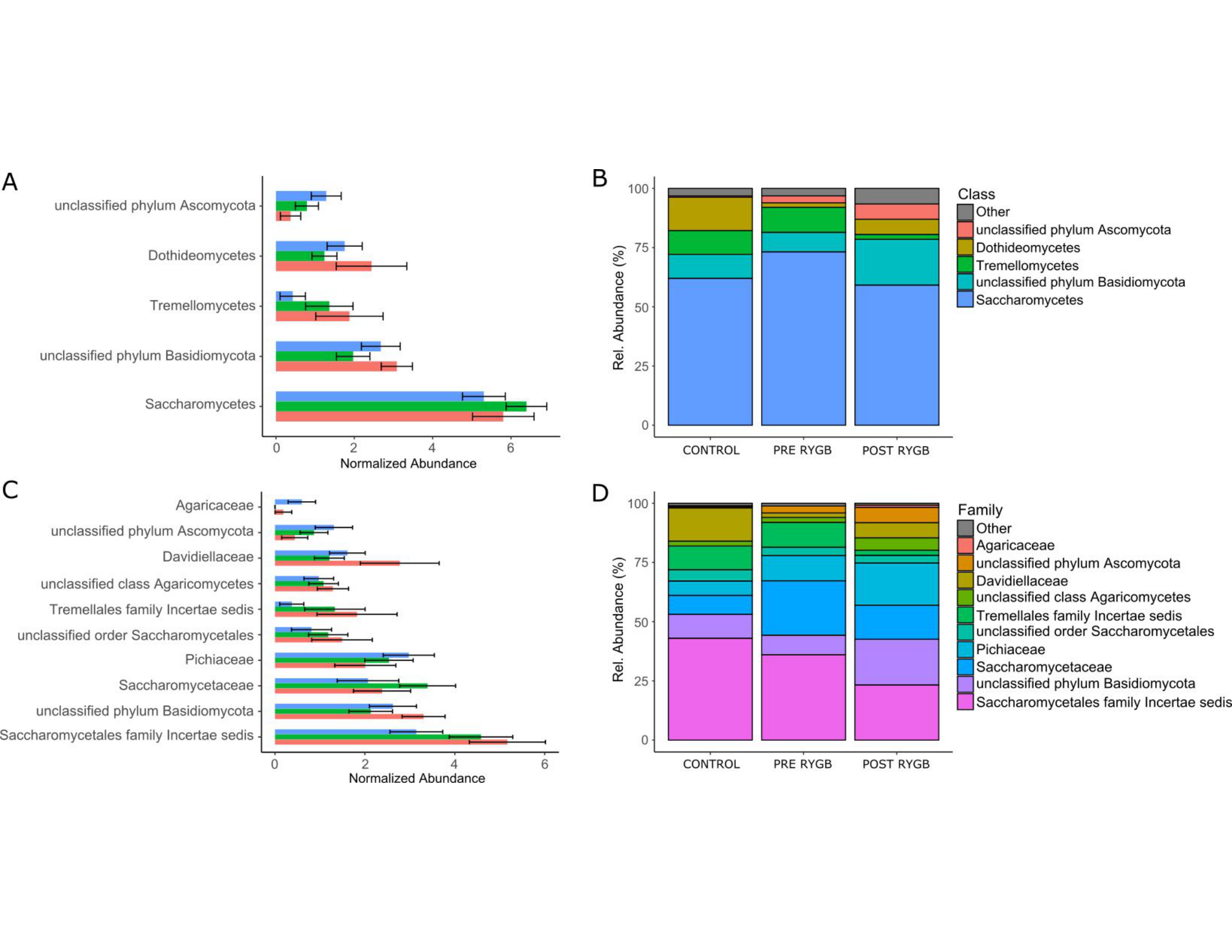

Supplement: S2 Fig — Minor fungi are grouped under ‘Other’. There were no significant differences between subject groups. (TIFF) [file pone.0236936.s003.tiff]
